# Acute Abdomen Secondary to Incarcerated Umbilical Hernia after Treatment of Massive Cirrhotic Ascites

**DOI:** 10.1155/2013/948172

**Published:** 2013-03-10

**Authors:** Hiang Keat Tan, Pik Eu Chang

**Affiliations:** ^1^Department of Gastroenterology and Hepatology, Singapore General Hospital, Outram Road, Singapore 169608; ^2^Duke-National University of Singapore Graduate Medical School Singapore, 8 College Road, Singapore 169857

## Abstract

Umbilical herniation is common in patients with liver cirrhosis and ascites. Rarely, they suffer from incarceration and strangulation of the umbilical hernia after treatment of ascites. We report 3 cases of umbilical hernia incarceration following removal of massive ascites with different treatment modalities. Physicians managing this group of patients should be aware of this rare and potentially fatal complication.

## 1. Introduction

Ascites is a common complication of liver cirrhosis. There is an increased incidence of umbilical hernia in patients with liver cirrhosis and ascites [1]. Most patients remain asymptomatic and rarely develop complications such as leakage, ulceration, spontaneous rupture, or incarceration [2]. Incarceration of umbilical hernia in cirrhotics is rare but is known to occur after treatment of ascites [2]. In this paper, we document development of incarcerated or symptomatic umbilical hernia in three patients being treated for ascites, two of which resulted in acute abdomen requiring emergency surgery.

## 2. Case Report

### 2.1. Case 1

This patient was a 59-year-old woman with Child's B cryptogenic liver cirrhosis complicated by massive diuretic-intractable ascites. Despite high doses of diuretics (spironolactone 300 mg OM and frusemide 100 mg OM), she continued to require frequent large volume paracentesis for symptomatic relief. Her diuretic dose could not be increased further due to development of diuretic-induced renal impairment and hyperkalemia, and she was managed with recurrent large volume paracentesis. Over a period of 4 months, she required monthly large volume paracentesis totaling 22 litres. She was noted to have an umbilical hernia but was asymptomatic. The patient was recommended but declined liver transplantation. 

In view of persistent diuretic-intractable ascites and frequent need for large-volume paracentesis, the patient underwent insertion of a transjugular intrahepatic portosystemic shunt (TIPS) for treatment of her refractory ascites. Baseline hepatic venous pressure gradient was markedly elevated at 22 mmHg. An abdominal coop loop was inserted the day prior to the TIPS insertion for complete drainage of ascites. TIPS was performed uneventfully with a reduction in the portosystemic gradient to 11 mmHg after TIPS.

**Figure 1:**
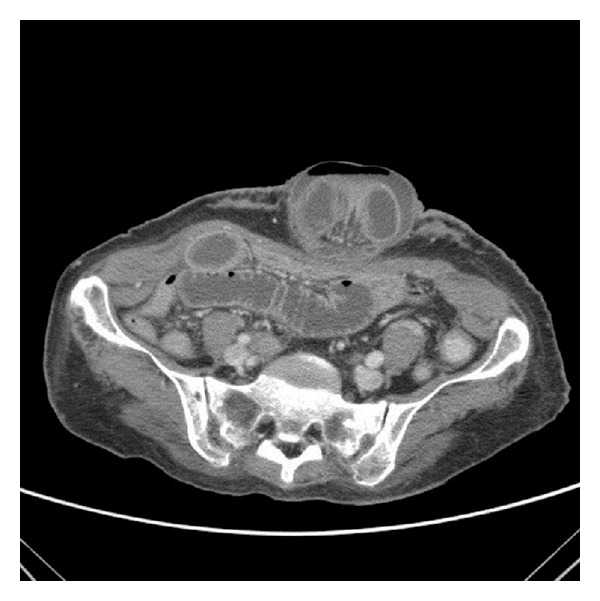
Umbilical hernia with dilated small bowel loop within and proximal to the hernia sac.

On the second day after the TIPS procedure, the patient complained of abdominal discomfort, nausea, and vomiting. Clinical examination revealed a tender irreducible umbilical hernia. Urgent computed tomography scan of the abdomen demonstrated an incarcerated umbilical hernia with dilated small bowel loops within and proximal to the hernia, with minimal residual ascites (Figure 1).

The patient underwent emergency laparotomy in which the incarcerated small bowel loop was found to be ischemic, requiring resection of an 8 cm length of small bowel. The patient recovered well postoperatively without any complication and is currently well. Her ascites remains satisfactorily controlled with spironolactone 150 mg OM and frusemide 80 mg OM without further need for large-volume paracentesis. 

### 2.2. Case 2

This patient was a 70-year-old Malay woman with severe idiopathic pulmonary hypertension complicated by cardiac cirrhosis and massive ascites. Transthoracic echocardiography revealed pulmonary arterial systolic pressure of 86 mmHg, severe tricuspid regurgitation, dilated inferior vena cava, and a left ventricular ejection fraction of 67%. She required monthly large-volume paracentesis for control of her massive ascites, draining 12 to 15 liters of ascitic fluid each time. She was on frusemide 40 mg twice daily. She had a pre-existing paraumbilical hernia which was noted more than a year prior but was never symptomatic. 

She was electively admitted for abdominal coop loop drainage of ascites in July 2011. Drainage catheter was inserted on admission, and over the next 2 days, 14.2 liters of ascitic fluid was drained. She complained of lower abdominal pain soon after the ascites had decreased significantly. Computed tomography scan of the abdomen confirmed the presence of small bowel obstruction with incarcerated paraumbilical hernia. She underwent emergency surgery. Intraoperatively, incarcerated paraumbilical loops were found and 10 cm of infarcted small bowel was resected. The patient made an uneventful postoperative recovery and was discharged on the 10th postoperative day. She still requires frequent large-volume paracentesis but with no further recurrence of the paraumbilical hernia. 

### 2.3. Case 3

This patient was a 56-year-old man with alcoholic liver cirrhosis with difficult to control ascites due to poor salt compliance and continued alcohol consumption. He was diagnosed in 2009 and was started on spironolactone 100 mg OM and frusemide 40 mg OM. The patient was maintaining well on spironolactone 50 mg OM from October 2009 until April 2011 when the diuretic had to be stopped because of severe hyponatremia and renal impairment. Clinically, the patient was cachectic with a distended abdomen and a reducible umbilical hernia. 

The patient was admitted in May 2011 for symptomatic tense ascites and had a large volume paracentesis, draining 16 liters of ascitic fluid under albumin cover. Diuretics were restarted during this admission, and he was advised on compliance to dietary sodium restriction and complete cessation of alcohol. He required 2 further sessions of large-volume paracentesis in June and July 2011. The dose of spironolactone was gradually increased to 175 mg OM in addition to frusemide 40 mg OM with no further drop in serum sodium levels.

The patient's ascites improved with optimization of diuretics and compliance to low salt diet. During a routine clinic visit in September 2011, the patient complained of abdominal pain. By this time, the ascites had improved and he only had mild ascites. On examination, the previously reducible umbilical hernia was irreducible and erythematous. He was admitted for further management, and the 4 × 4 cm umbilical hernia was successfully reduced manually after 10 minutes of manipulation. The patient was then referred to the hepatobiliary surgeon who scheduled him for an elective surgical repair of the umbilical hernia 2 weeks later. The patient underwent an uneventful herniorrhaphy and is currently well on spironolactone 75 mg OM with satisfactory control of the ascites.

## 3. Discussion

Umbilical herniation occurs in up to 20% of patients with advanced liver cirrhosis and ascites [1]. Increased intra-abdominal pressure as a result of ascites results in thrusting of the peritoneum forward through the umbilical ring leading to herniation of the skin envelope above the abdominal wall [2]. Patients with advanced liver cirrhosis often have protein-calorie malnutrition leading to abdominal wall muscle wasting, and this contributes to the increased incidence of umbilical hernia in this group of patients [2]. Portosystemic collaterals may also result in recanalization of the umbilical vein, and this contributes to further weakening of the abdominal wall and herniation of the umbilicus [3]. 

In patients with distended abdomen due to large volume ascites, the fascial defect in the umbilical hernia remains widely patent, and therefore incarceration is unlikely. The presence of large volume ascites within the abdominal cavity provides buoyancy to the bowel contents within the hernia, thus preventing strangulation. Following the rapid removal of ascites, there is decreased tension on the umbilical hernia ring, with the subsequent decrease in diameter and trapping of hernia sac contents. Strangulation of umbilical hernia following resolution of ascites has been reported to occur with diuretics alone [4, 5], after large volume paracentesis [5, 6], transjugular-intrahepatic portosystemic shunt [7], and peritoneovenous shunt [5, 8].

In our series, incarceration of the umbilical hernia occurred rapidly in two patients, occurring within days of resolution of ascites following TIPS and large volume paracentesis, respectively, resulting in strangulation and small bowel infarction. In patients managed with diuretics and salt restriction, control or resolution of ascites usually occurs at a much slower rate, and therefore symptomatic or incarceration of the umbilical hernia tends to occur weeks to months later after starting therapy, as seen in the third patient and in other case reports [4].

Historically, patients with liver cirrhosis and umbilical hernia are often managed conservatively because of high postoperative mortality and morbidity with high postoperative recurrence [9]. In patients whose ascites can be controlled by medical therapy, surgical herniorrhaphy can be performed safely with low mortality and morbidity [2]. Five to 10% of ascitic patients per year become refractory to standard medical therapy [10], and these patients are at risk of spontaneous rupture of the umbilical hernia and infection. Spontaneous rupture of umbilical hernia in patients with liver cirrhosis has been associated with mortality as high as 30% [11]. Mortality in this group of patients is often due to variceal haemorrhage, renal failure, or sepsis from peritonitis [11].

Optimal management of patients with liver cirrhosis and umbilical hernia remains uncertain. In patients who are candidates for liver transplantation, a more conservative approach while awaiting definitive surgical repair concurrent with liver transplantation is appropriate [12]. In others, a more aggressive approach involving preoperative TIPS [13] and optimization of nutrition [14] followed by semiemergent surgical repair has been shown to have a lower than expected mortality and morbidity. A conservative approach of operating only in patients with complications from the umbilical hernia has a significantly poorer outcome [12].

In conclusion, umbilical herniation is common in patients with large symptomatic ascites, and resolution of ascites can result in incarceration and strangulation of the umbilical hernia. Physicians managing such patients should be aware of this uncommon complication and should have a high index of suspicion for the prompt diagnosis and treatment of this entity. In patients undergoing TIPS or large-volume paracentesis, physicians should perform external reduction of umbilical hernias before the procedure.
